# Optimization of Initial Dose Regimen for Sirolimus in Pediatric Patients With Lymphangioma

**DOI:** 10.3389/fphar.2021.668952

**Published:** 2021-11-08

**Authors:** Xiao Chen, Dongdong Wang, Guangfei Wang, Yidie Huang, Xin Yu, Jinmiao Lu, Xiaowen Zhai, Hong Xu, Zhiping Li

**Affiliations:** ^1^ Department of Pharmacy, National Children’s Medical Center, Children’s Hospital of Fudan University, Shanghai, China; ^2^ Department of Hematology and Oncology, National Children’s Medical Center, Children’s Hospital of Fudan University, Shanghai, China; ^3^ Department of Nephrology, National Children’s Medical Center, Children’s Hospital of Fudan University, Shanghai, China

**Keywords:** optimization, initial dose regimen, sirolimus, pediatric, lymphangioma

## Abstract

Sirolimus is an effective oral treatment for pediatric patients with lymphangioma. The present clinical study in 15 children (0.12–16.39 years of age) examines the effects of underlying factors on sirolimus concentrations through application of a population pharmacokinetic model. Using Monte Carlo simulation, an initial dose regimen for sirolimus in pediatric patients with lymphangioma is presented. It is found that the lower the body weight, the higher the clearance rate and sirolimus clearances are 0.31–0.17 L/h/kg in pediatric patients with lymphangioma whose weights are 5–60 kg, respectively. The doses of sirolimus, 0.07, 0.06, 0.05 mg/kg/day are recommended for weights of 5–10, 10–24.5 and 24.5–60 kg in children with lymphangioma. This study is the first to establish a population pharmacokinetic model for sirolimus and to recommend initial doses in pediatric patients with lymphangioma. Large scale, prospective studies are needed in the future.

## Introduction

Lymphangioma, which is also called lymphatic malformation, is rare and benign anomaly derived from the defective embryological development of the primordial lymphatic structure (Amodeo et al., 2017b). Disease progress includes the progressive growth of the lymphangioma leading to compression of the adjacent structures (Reinglas et al., 2011). On account of their permeative growth throughout all tissue layers, treatment is often challenging and controversial (Laforgia et al., 2016; Amodeo et al., 2017b). Historically, surgical excision is one choice of treatment considered; however, less invasive therapy is now preferred (Amodeo et al., 2017b).

At present, it has been reported that sirolimus is an oral effective medication for lymphangioma (Reinglas et al., 2011; Amodeo et al., 2017a; Amodeo et al., 2017b). However, sirolimus has a narrow therapeutic window and its considerable inter- and intra-individual pharmacokinetic variabilities make it difficult to design an initial dosing schedule (Chen et al., 2020; Cheng et al., 2020; Wang et al., 2020), especially in pediatric patients with lymphangioma. Hence, the present study aims to investigate the effects of underlying factors on clinical sirolimus concentrations by building up a population pharmacokinetic model, and to recommend initial dose regimen for sirolimus in pediatric patients with lymphangioma using Monte Carlo simulation.

## Methods

### Patients

Pediatric patients with lymphangioma from May 2016 to December 2020 at the Children’s Hospital of Fudan University (Shanghai, China) were collected, retrospectively. The study was approved by the Research Ethics Committee of Children’s Hospital of Fudan University [Ethical code: (2019) 019]. Because the study was retrospective, it was approved by the ethics committee of our hospital without the need for written informed consent. The inclusion criteria were 1) treated with sirolimus, and 2) recorded therapeutic drug monitoring (TDM) for sirolimus. Demographic data of patients were collected, such as gender, age, weight, albumin, alanine transaminase, aspartate transaminase, creatinine, urea, total protein, total bile acid, direct bilirubin, total bilibrubin, hematocrit, hemoglobin, mean corpuscular hemoglobin, mean corpuscular hemoglobin concentration.

### TDM

The Emit 2000 Sirolimus Assay (Siemens Healthcare Diagnostics Inc.) with range of linear response, 3.5–30 ng/ml, whose values of inter-assay variability [coefficient of variation (CV%)] < 4.0%, and values of intra-assay CV (%) < 6.2%, were used to measure sirolimus concentrations.

### Population Pharmacokinetic Model

The non-linear mixed-effects modeling software, NONMEM (edition 7, ICON Development Solutions, Ellicott City, MD, USA) and a first-order conditional estimation method with interaction (FOCE-I method) were used to establish the population pharmacokinetic model of sirolimus in pediatric patients with lymphangioma. The apparent oral clearance (CL/F), volume of distribution (V/F), and absorption rate constant, Ka (fixed at 0.485/ h), were included in pharmacokinetic parameters (Wang et al., 2020).

#### Random Effect Model

Equation 1 showed the inter-individual variability:

$$M_{i}\mathrm{=TV}\left(M\right)\times \exp\left(\eta_{i}\right)$$ (1)

M_i_, the individual parameter value; TV(M), the typical individual parameter value; η_i_, symmetrical distribution, which was random term with zero mean and variance omega^2 (ω^2^).

Equation 2 showed random residual variability

$$N_{i}=L_{i}+\varepsilon_{1}$$ (2)

N_i_, the observed concentration; L_i_, the individual predicted concentration; ε_1_, symmetrical distribution, which was random term with zero mean and variance sigma^2 (σ^2^).

#### Covariate Model

Equation 3 showed the relations of pharmacokinetic parameters with weight:

$$X_{i}=X_{\mathrm{std}}\times {\left(Y_{i}/Y_{\mathrm{std}}\right)}^{power}$$ (3)

X_i_, the *i*th individual parameter; Y_i_, the *i*th individual weight; Y_std_, the standard weight of 70 kg; X_std_, the typical individual parameter, whose weight was Y_std_; power, the allometric coefficient: 0.75 for the CL/F and 1 for the V/F(Anderson and Holford, 2008).

Equation 4 and Equation 5 showed continuous or categorical covariates with pharmacokinetic parameters, respectively:

$$Z_{i}\mathrm{=TV}\left(Z\right)\times {\left(\mathrm{Co}v_{i}\mathrm{/Co}v_{\mathrm{median}}\right)}^{\theta }$$ (4)

$$Z_{i}\mathrm{=TV}\left(Z\right)\times \theta^{\mathrm{Covi}}$$ (5)

Z_i_, the individual parameter value; TV(Z), the typical individual parameter value; θ, the parameter to be estimated; Cov_i_, the covariate of the *i*th individual; Cov_median_, the population median for the covariate.

Objective function value (OFV) changes were used as the covariate inclusion criteria, whose decrease greater than 3.84 (*p* < 0.05) was considered sufficient for inclusion in the base model. An increase greater than 6.63 (*p* < 0.01) was considered sufficient for significance in the final model.

### Model Evaluation

The goodness-of-fit plots of model, conditional weighted residuals (WRES) *vs.* time after the start therapy, observations *vs.* population predictions, observations *vs.* individual predictions, conditional WRES *vs.* population predictions, conditional WRES *vs.* individual predictions were used to evaluate model visualization. Distribution of weighted residuals for model, density *vs.* weighted residuals, and quantilies of weighted residuals *vs.* quantilies of normal, were used to evaluate the model distribution. Prediction-corrected visual predictive check (VPC) of model were used to evaluate model predictability. Additionally, 1,000 time bootstraps were used to evaluate model stability.

### Simulation

The target concentration window of sirolimus in pediatric patients with lymphangioma was 5-15 ng/ml(Reinglas et al., 2011; Amodeo et al., 2017b; Lagreze et al., 2019), and initial doses were simulated using Monte Carlo method. In the present study, 1,000 virtual pediatric patients were simulated in seven weight groups (5, 10, 20, 30, 40, 50, and 60 kg) for ten dosages (0.01, 0.02, 0.03, 0.04, 0.05, 0.06, 0.07, 0.08, 0.09, and 0.10 mg/kg/day), respectively. The sirolimus regimen was divided evenly into two doses per day. The evaluation criteria were the probabilities of achieving concentrations within the target window.

## Results

### Patients

A total of 15 pediatric patients with lymphangioma are included in the present study, 12 boys and 3 girls, aged from 0.12 to 16.39 years old and weighted from 4 to 54 kg. Demographic data of pediatric patients with lymphangioma are shown in Table 1.

**TABLE 1:** Demographic data of patients.

Characteristic	Mean ± SD	Median (range)
Gender (boys/girls)	12/3	—
Age ( years)	7.29 ± 3.11	6.80 (0.12–16.39)
Weight ( kg)	22.27 ± 9.87	21.00 (4.00–54.00)
Albumin ( g/L)	37.82 ± 7.52	38.50 (21.60–46.40)
Alanine transaminase ( IU/L)	22.41 ± 23.33	15.45 (6.00–83.60)
Aspartate transaminase ( IU/L)	40.61 ± 32.19	36.00 (16.00–133.80)
Creatinine ( μmol/L)	32.36 ± 16.18	29.00 (16.00–76.00)
Urea ( mmol/L)	3.89 ± 1.18	3.70 (2.20–5.70)
Total protein ( g/L)	63.88 ± 15.90	68.50 (30.80–91.90)
Total bile acid ( μmol/L)	5.03 ± 2.63	5.30 (1.20–10.30)
Direct bilirubin ( μmol/L)	1.63 ± 0.86	1.60 (0.60–3.00)
Total bilibrubin ( μmol/L)	4.77 ± 2.22	4.40 (1.80–9.40)
Hematocrit (%)	33.19 ± 5.26	32.40 (23.00–43.40)
Hemoglobin ( g/L)	109.26 ± 20.99	104.00 (77.00–156.00)
Mean corpuscular hemoglobin (pg)	26.34 ± 2.58	26.00 (22.00–31.00)
Mean corpuscular hemoglobin concentration ( g/L)	328.00 ± 16.27	331.00 (292.00–359.00)

### Modeling

In the final model, weight is included as a covariant and the final model is shown below:

$$\mathrm{CL/F=11}\mathrm{.3}\times {\left(\mathrm{weight/70}\right)}^{0.75}$$ (6)

V/F=388×(Weight/70) (7)

CL/F, apparent oral clearance; V/F, apparent volume of distribution.

### Evaluation

**FIGURE 1:**
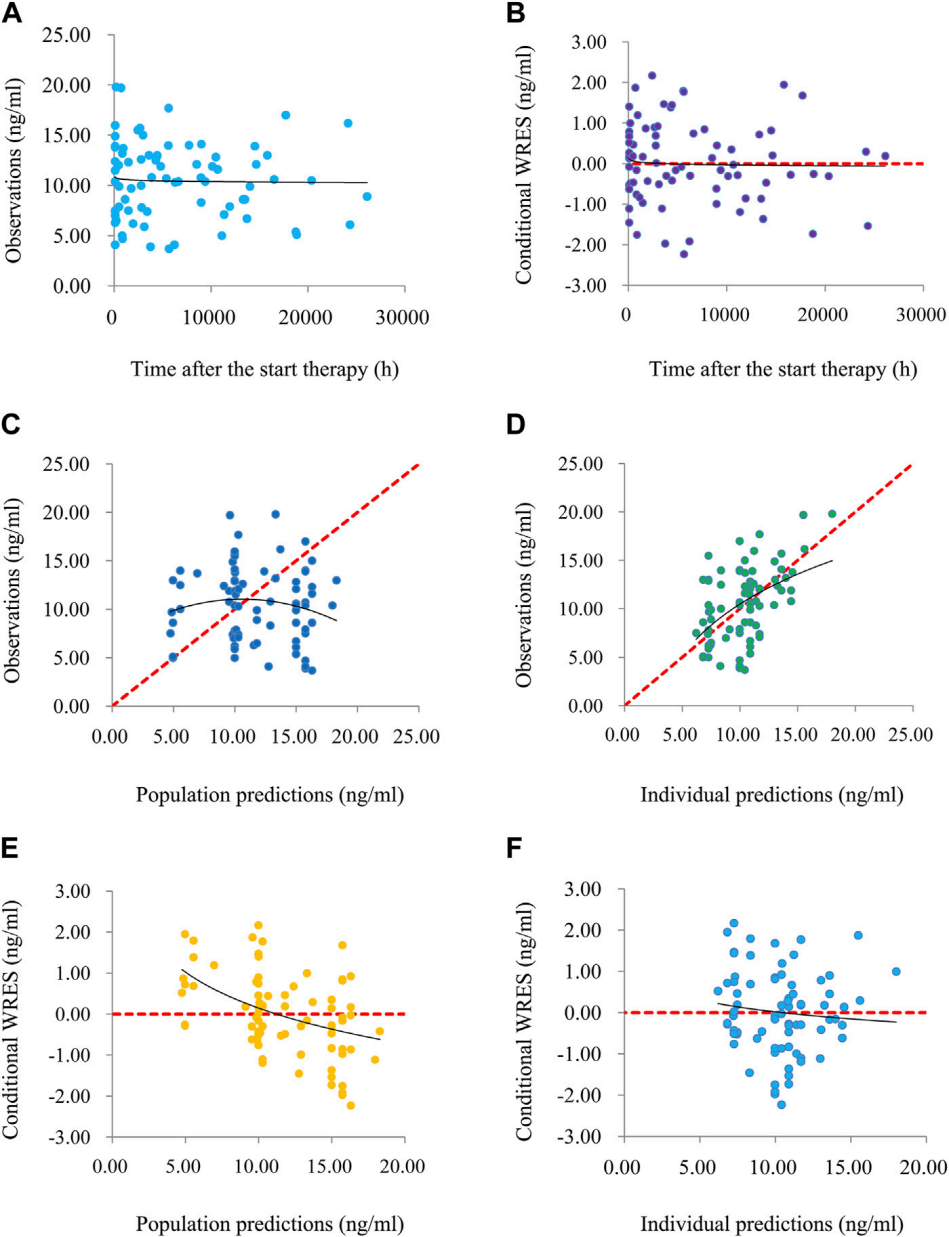
The goodness-of-fit plots of model. **(A)** Time-concentration diagram. **(B)** Conditional weighted residuals (WRES) *vs.* time after the start therapy. **(C)** Observations *vs.* population predictions. **(D)** Observations *vs.* individual predictions. **(E)** Conditional WRES *vs.* population predictions. **(F)** Conditional WRES *vs.* individual predictions.

**FIGURE 2:**
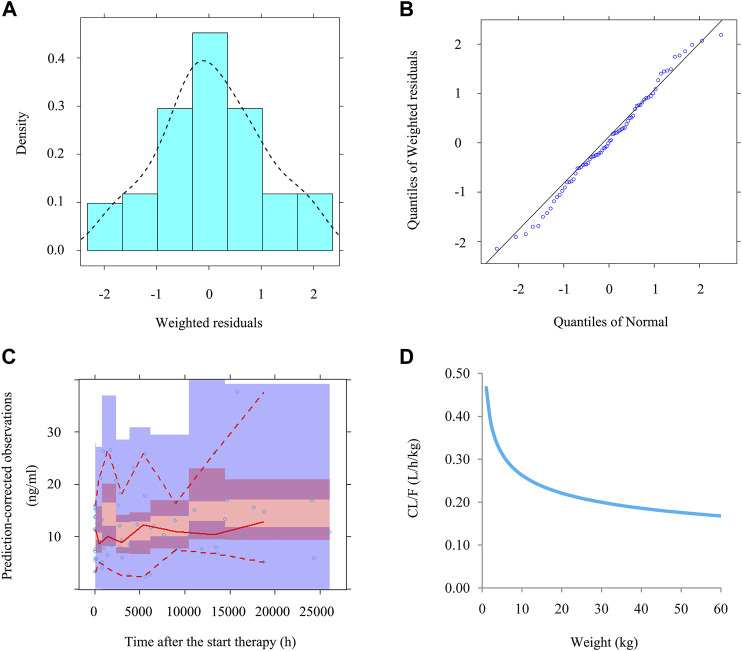
The evaluation of normality and VPC. **(A)** Density *vs.* weighted residuals. **(B)** Quantilies of weighted residuals *vs.* quantilies of normal. **(C)** Prediction-corrected visual predictive check (VPC) of model. The middle solid line represents the median of the prediction-corrected concentrations. The lower and upper dashed lines are the 2.5 and 97.5th percentiles of the prediction-corrected concentrations. **(D)** Sirolimus apparent clearance rate of pediatric patients with different weight.

Time-concentration diagrams and model evaluations are shown in Figure 1. Figure 1A displays observations *vs.*time after the start therapy, and Figures 1B–F show goodness-of-fit plots of model, in which a good model fitting effect is demonstrated. Figures 2A,B demonstrate the distribution of weighted residuals, following the normal distribution. Figure 2C shows prediction-corrected VPC on the model confirming that most of the observed sirolimus concentrations are within the 95% prediction intervals from the simulation data, and the prediction-corrected sirolimus concentrations are well predicted by the final model. In Table 2, the parameter estimates of the final model are within 95% confidence interval of 1,000 bootstraps, showing that the model is reliable.

**TABLE 2:** Parameter estimates of final model and bootstrap validation.

Parameter	Estimate	SE (%)	Bootstrap (n = 1,000)
			95% confidence interval
CL/F (L/h/70 kg)	11.3	9.5	(0.7, 13.4)
V/F (L/70 kg)	388	41.8	(1, 648)
Ka (h^**−**^1)	0.485 (fixed)	—	—
ωCL/F	0.303 (13.7%)	18.5	(0.102, 0.355)
σ1 ( ng/ml)	3.578	3.7	(3.056, 3.808)

95% confidential interval was displayed as the 2.5 and 97.5th percentile of bootstrap estimates. CL/F, apparent oral clearance (L/h); V/F, apparent volume of distribution (L); Ka, absorption rate constant (h^−1^); ω_CL/F_, inter-individual variability of CL/F; σ_1_, sigma, residual variability, additive error.

### Simulation

**FIGURE 3:**
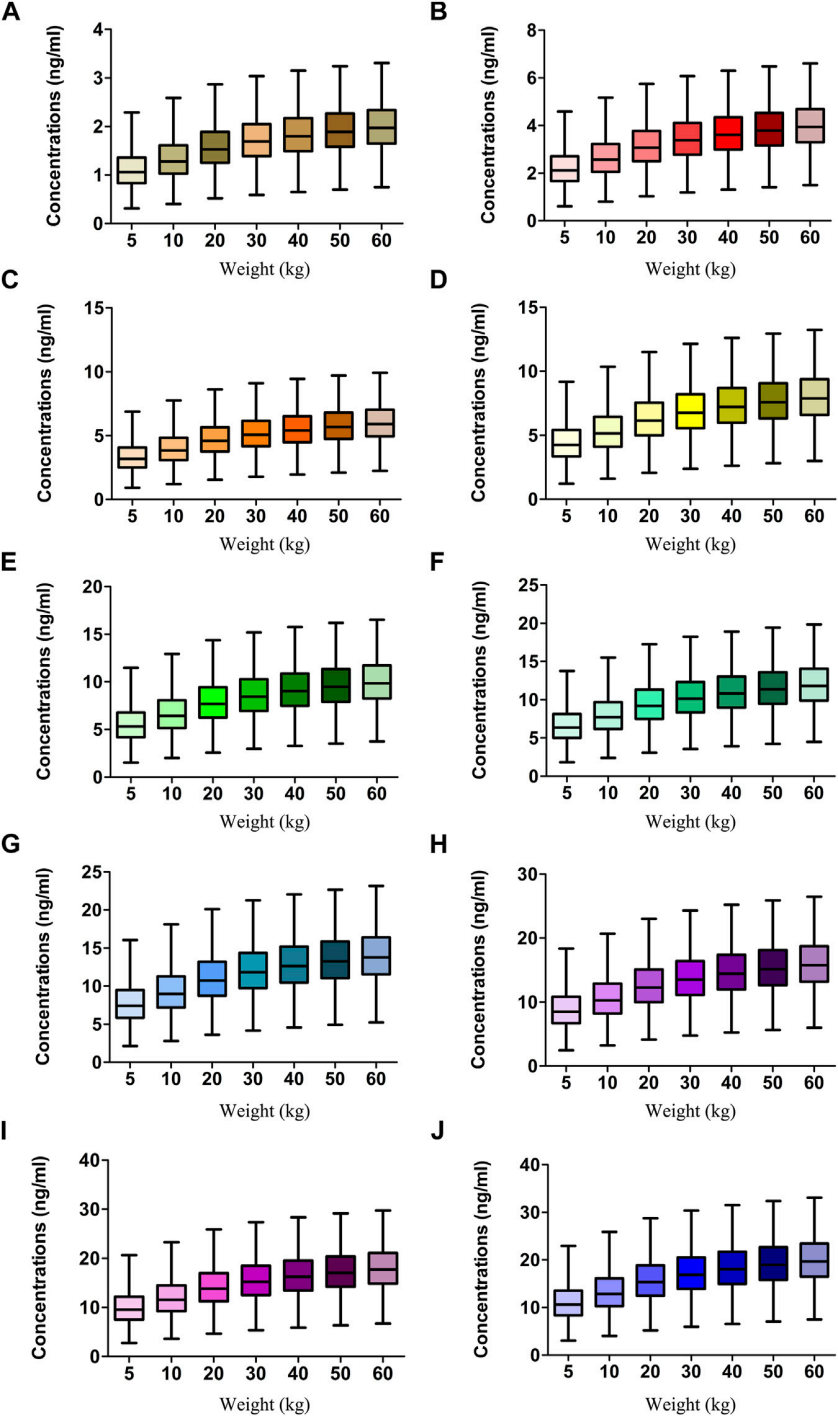
Simulation of sirolimus concentrations. **(A)** 0.01 mg/kg/day sirolimus dose. **(B)** 0.02 mg/kg/day sirolimus dose. **(C)** 0.03 mg/kg/day sirolimus dose. **(D)** 0.04 mg/kg/day sirolimus dose. **(E)** 0.05 mg/kg/day sirolimus dose. **(F)** 0.06 mg/kg/day sirolimus dose. **(G)** 0.07 mg/kg/day sirolimus dose. **(H)** 0.08 mg/kg/day sirolimus dose. **(I)** 0.09 mg/kg/day sirolimus dose. **(J)** 0.10 mg/kg/day sirolimus dose. The sirolimus regimen was splited evenly into two dosages a day.

**FIGURE 4:**
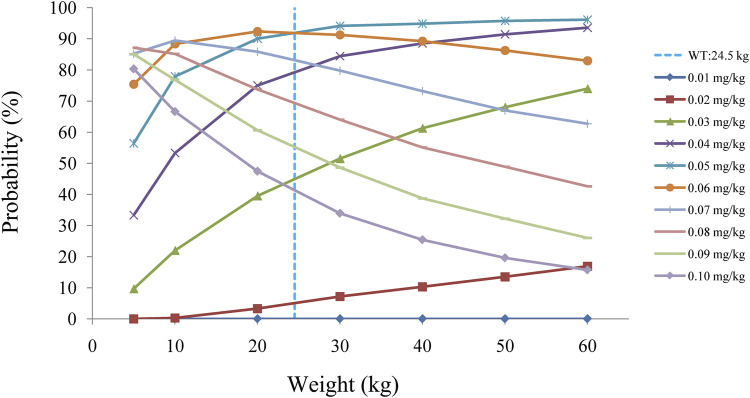
Probability to achieve the target concentrations.

**FIGURE 5:**
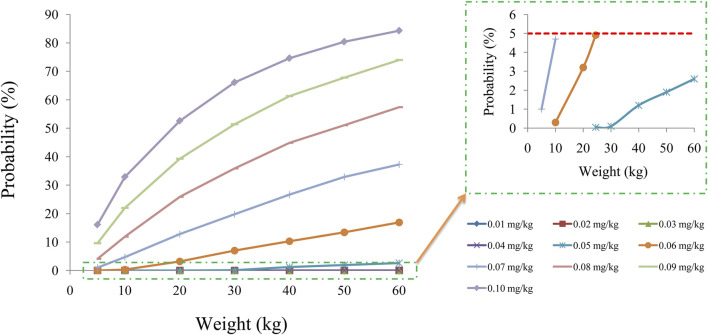
Probability to exceed the upper limit of the target concentrations.

Figure 2D displays the clearance rate of sirolimus in pediatric patients with lymphangioma, and we find that the lower the body weight, the higher the clearance rate and sirolimus clearances are 0.31-0.17 L/h/kg in pediatric patients with lymphangioma, whose weight are 5–60 kg, respectively. Initial dose simulations are shown in Figure 3, and Figures 3A–J are for ten dosages (0.01, 0.02, 0.03, 0.04, 0.05, 0.06, 0.07, 0.08, 0.09, and 0.10 mg/kg/day), respectively. The evaluation criteria are the probabilities of achieving target concentration window, and the doses of sirolimus, 0.07, 0.06, 0.05 mg/kg/day are recommended for weights of 5–10, 10–24.5, and 24.5–60 kg in pediatric patients with lymphangioma, respectively, which are shown in Figure 4 and Table 3. In addition, Figure 5 shows the security aspect and at the current recommended doses, the probabilities to exceed the upper limit of the target concentrations are less than 5%, indicating relatively high security.

**TABLE 3:** Initial dosage recommendation of sirolimus.

Body weight ( kg)	Dose (mg/kg/day) **^a^**	Probability to achieve the target concentrations (%)	Probability to exceed the upper limit of the target concentrations (%)
5–10	0.07	85.2–89.4	1.0–4.7
10–24.5	0.06	88.3–92.3	0.3–4.91
24.5–60	0.05	91.1–96.1	0.045–2.6

a Split evenly into two doses a day.

## Discussion

Sirolimus, the mammalian target of rapamycin (mTOR) inhibitor, is an immunosuppressive and antitumor drug. The mTOR signaling is now known to be linked to vascular development, whose activation can increase the expression of vascular endothelial growth factor A and C, crucial regulators of angiogenesis and lymphangiogenesis respectively, promoting vascular proliferation and cell growth (Amodeo et al., 2017b). Sirolimus can block the mTOR signaling and further inhibit abnormal vascular proliferation, reducing the production of vascular endothelial growth factor and responsiveness of its receptors (Neuhaus et al., 2001; Huber et al., 2007; Kobayashi et al., 2007; Lee and Hung, 2007; Hammill et al., 2011; Shibuya, 2011).

Sirolimus was first used as an immunosuppressant agent to augment prevention of rejection in solid organ transplantation, following kidney transplant (Pinto-Leite et al., 2009). However, at present, many studies have reported that sirolimus can be used as an anticancer treatment (Wan and Helman, 2007; Cloughesy et al., 2008). For example, sirolimus is a potentially effective treatment option for patients with hormone receptor-positive advanced breast cancer (Yi et al., 2020). The combination of pemetrexed and sirolimus is active in previously treated non-small cell lung cancer (Komiya et al., 2019). Combination of metformin and sirolimus show the most potent inhibition in all colon cancer cell lines (Mussin et al., 2017). Switching from calcineurin inhibitors to sirolimus has an antitumoral effect among kidney-transplant recipients with previous squamous-cell carcinoma (Euvrard et al., 2012). In addition, it has been also reported that sirolimus is an oral effective medication for lymphangioma (Reinglas et al., 2011; Amodeo et al., 2017a; Amodeo et al., 2017b).

Lymphangioma is an uncommon, benign malformations of the lymphatic system that can occur anywhere on the skin and mucous membranes (Miceli and Stewart, 2020). Lymphangioma can be categorized as deep or superficial based on the depth and size of the abnormal lymphatic vessels or as congenital or acquired (Miceli and Stewart, 2020). In recent years there have been increasing reports on the treatment of lymphangioma with sirolimus (Amodeo et al., 2017b; Lagreze et al., 2019). However, the adverse effects of sirolimus have limited its clinical application to some extent. Its most significant adverse effects are anemia, anorexia, asthenia, edema, elevated alkaline phosphatase, elevated serum creatinine, hyperglycemia, hyperlipemia, hypertriglyceridemia, hypophosphatemia, lymphopenia, nausea, rash, thrombocytopenia (Amodeo et al., 2017b). In addition, rare but potentially serious adverse effects of sirolimus have also been found such as cardiac disorders and infections (Klumpen et al., 2010; Ji et al., 2017).

The majority of adverse effects are related to the concentrations of sirolimus, and in clinical practice, TDM is used to measure sirolimus concentrations. The following doses of sirolimus are regulated on the strength of concentrations. However, with narrow therapeutic range and PK variabilities it is hard to project an initial dosing schedule for use in pediatric patients with lymphangioma. Fortunately, the optimum initial regimen can be recommended by the combination of population pharmacokinetics and Monte Carlo simulation. For example, Cheng *et al* reported the first study in pediatric: population pharmacokinetics of sirolimus and its application in Chinese children with immune cytopenia (Cheng et al., 2020). Wang *et al* reported initial dosage recommendation for sirolimus in children with tuberous sclerosis complex (Wang et al., 2020). Chen *et al* reported initial dose recommendation for sirolimus in paediatric kaposiform haemangioendothelioma patients (Chen et al., 2020). Thus, our study aims to explore the effects of underlying factors on clinical sirolimus concentrations with a population pharmacokinetic model, and to recommend initial dose regimen for sirolimus in pediatric patients with lymphangioma.

In the present study, sirolimus clearance is affected by weight. It had been reported that there is a non-linear relationship between drug clearance and weight in pediatric patients, which may be well described by allometric scaling with the coefficients of 0.75 for clearance and 1 for volume (Anderson and Holford, 2008; Hao et al., 2018). In addition, we find that the lower the body weight, the higher the clearance rate. In other words, the smaller the child’s weight, the higher the dose required. Afterwards, the initial dosing protocol is simulated based on the final model, the target concentration window of sirolimus in pediatric patients with lymphangioma is 5–15 ng/ml (Reinglas et al., 2011; Amodeo et al., 2017b; Lagreze et al., 2019). The doses of sirolimus, 0.07, 0.06, and 0.05 mg/kg are recommended respectively for weights of 5–10, 10–24.5, and 24.5–60 kg in pediatric patients with lymphangioma. “Children are NOT small adults”, and our innovation lies in that the model we use to simulate the dose is taken from the population of pediatric patients with lymphangioma, so that the model can more accurately reflect the real situation of sirolimus in pediatric patients with lymphangioma, instead of referring to adult doses again.

The metabolism of sirolimus has been shown to be influenced by *CYP3A4* and *CYP3A5* in adults (Tamashiro et al., 2017; Zhang et al., 2017). However, no significant effect such as this was found in children. For example, *CYP3A4* and *CYP3A5* polymorphism did not significantly affect sirolimus clearance rates in children with tuberous sclerosis complex (Wang et al., 2020). Also, CYP3A genotypes were not included in population pharmacokinetics of sirolimus for children with recurrent solid tumours or complicated vascular anomalies (Mizuno et al., 2017a; Mizuno et al., 2017b). Thus, our study do not further explore the impacts from *CYP3A4* and *CYP3A5* genotypes. Furthermore, no routine tests are performed for sirolimus pharmacogenomics in clinical therapy. The dose recommendation on body weight was considered to be more convenient and practical in a clinical setting (Wang et al., 2020). And there are evidences to support this conclusion. For example, in Wang *et al*’s study, only body weight was fixed in the covariate model (Wang et al., 2020). And in other drug studies, such as in Niu *et al*’s study, population pharmacokinetics and dosing regimen optimisation of lopinavir in Chinese adults infected with HIV, only body weight was included into model (Niu et al., 2019).

However, due to the low incidence of lymphangioma, the present study has objective difficulties in the collection of children patients, resulting in the limited number of pediatric patients with lymphangioma. A total of 15 pediatric patients with lymphangioma are included in the present study. Given the low incidence of the disease, our small number of patients may be objectively acceptable. Because similar studies with smaller numbers have been reported, for example, in Mizuno *et al*’s study, total 19 patients were included (Mizuno et al., 2017a). In addition, in Wang *et al*’s study, total 15 patients were included (Wang et al., 2020). Meanwhile, similarly, in Mizuno *et al*’s study, the covariate analysis inclusion of age and sex did not significantly improve the temsirolimus model (Mizuno et al., 2017a). In Wang *et al*’s study, only body weight was fixed in the covariate model, and recommending doses were based on body weight (Wang et al., 2020). As for the limited sample size, these covariates should still be evaluated in an adequately powered future study. But then again, in the current limited number of children, body weight as a covariable can better fit the clinical data in the real world, which to some extent can provide beneficial reference for solving the missing individual drug administration of sirolimus in pediatric patients with lymphangioma, and better lay the foundation for the following large-scale clinical research. Even so, studies with larger sample sizes need to be further studied in the future.

## Conclusion

This study has limitations because of the rarity of lymphangioma and the use of retrospective observations. However, it is the first report of a population pharmacokinetic model for sirolimus with recommendations for initial dosing in pediatric patients with lymphangioma. Large scale and prospective studies are needed in the future to confirm and refine the preliminary findings presented here.

## Data Availability

The raw data supporting the conclusions of this article will be made available by the authors, without undue reservation.
