# Supplementary figures and images for: Optimization of Initial Dose Regimen for Sirolimus in Pediatric Patients With Lymphangioma
Source: Front Pharmacol. 2021 Nov 8;12:668952. doi: 10.3389/fphar.2021.668952 (PMC8606893; doi:10.3389/fphar.2021.668952)

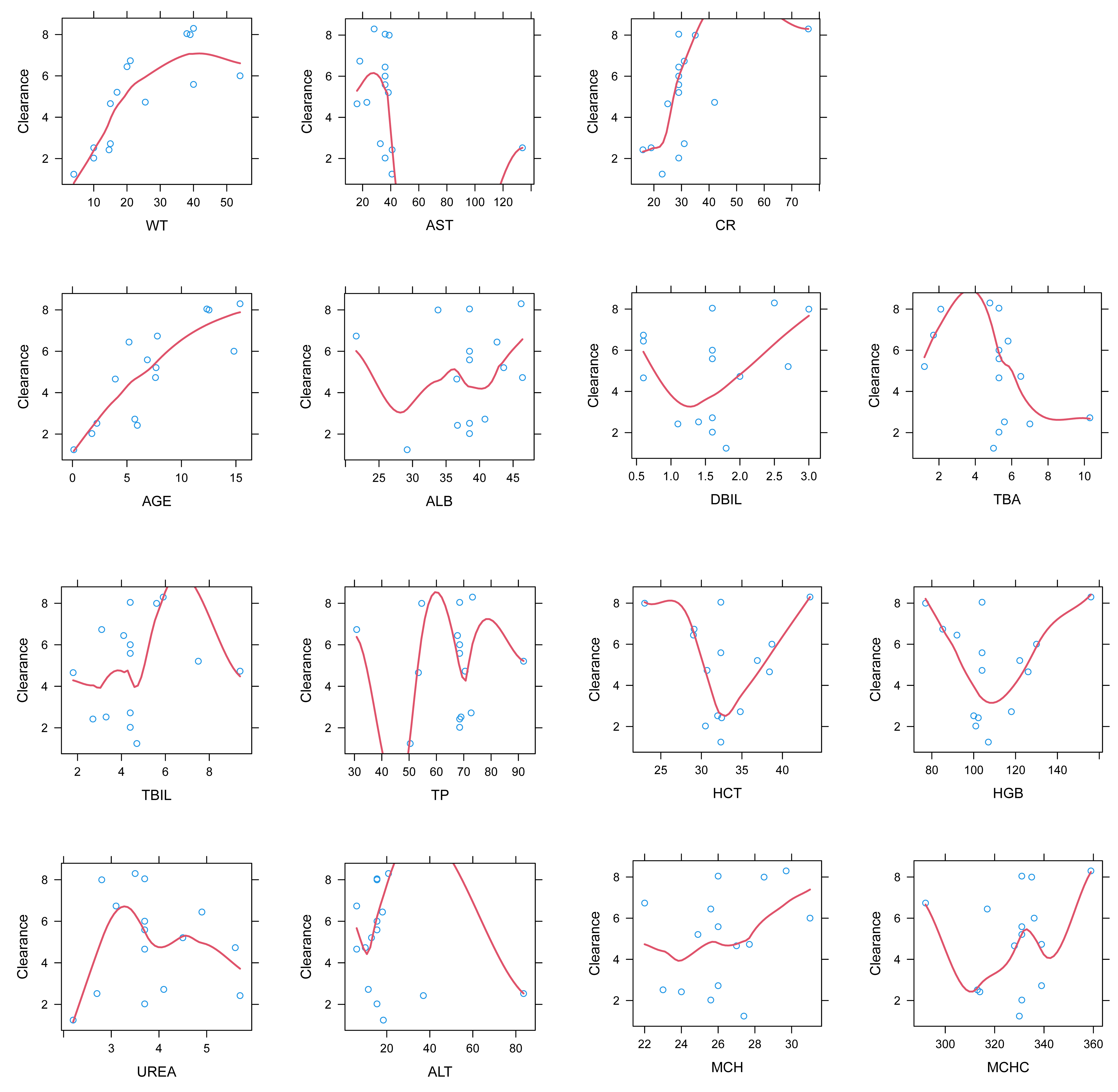

Supplement: Supplementary file 1 [file Image1.TIF]
